# Optimization and Analytical Behavior of Electrochemical Sensors Based on the Modification of Indium Tin Oxide (ITO) Using PANI/MWCNTs/AuNPs for Mercury Detection

**DOI:** 10.3390/s20226502

**Published:** 2020-11-14

**Authors:** Noor Aini Bohari, Shafiquzzaman Siddiquee, Suryani Saallah, Mailin Misson, Sazmal Effendi Arshad

**Affiliations:** 1 Biotechnology Research Institute, University Malaysia Sabah, Jalan UMS, Kota Kinabalu 88400, Sabah, Malaysia; noorainibohari@gmail.com (N.A.B.); suryani@ums.edu.my (S.S.); mailin@ums.edu.my (M.M.); 2 Faculty of Science and Natural Resources, University Malaysia Sabah, Jalan UMS, Kota Kinabalu 88400, Sabah, Malaysia; sazmal@ums.edu.my

**Keywords:** mercury, cosmetic, cyclic voltammetry (CV), differential pulse voltammetry (DPV), electrochemical sensor

## Abstract

In the present study, indium tin oxide (ITO) was used as a transparent working electrode for the development of an electrochemical sensor for the detection of mercury (II) ions (Hg^2+^). The electrode was modified by direct electrodeposition of polyaniline (PANI), multiwalled carbon nanotubes (MWCNTs) and gold nanoparticles (AuNPs) followed by optimization of the analyte and operating conditions, aiming to improve the selectivity, sensitivity and reliability of the electrode for mercury detection. Successful immobilization of the PANI and nanomaterials (MWCNTs and AuNPs) on the ITO electrode was confirmed by Scanning Electron Microscope (SEM), Energy Dispersive X-ray (EDX) and Fourier Transform Infrared Spectroscopy (FTIR) analyses. The optimum conditions for mercury detection using the modified ITO electrode were pH 7.0 of Tris-HCl buffer (50 mM) in the presence of 1 mM methylene blue (MB) as a redox indicator, a scan rate of 0.10 V·s^−1^ and a 70 s interaction time. The electrochemical behavior of the modified electrode under the optimized conditions indicated a high reproducibility and high sensitivity of mercury detection. It is therefore suggested that the PANI/MWCNT/AuNP-modified ITO electrode could be a promising material for the development of on-site mercury detection tools for applications in fields such as diagnostics, the environment, safety and security controls or other industries.

## 1. Introduction

Mercury has long been used as a bleaching agent in cosmetic products. This chemical acts by suppressing the production of melanin and by removing dead skin cells, resulting in a stereotypical slate-gray skin color. Nonetheless, it may cause skin rashes and scarring and reduce skin resistance to bacterial and fungal infections. Most importantly, being a neurotoxin, mercury exposure has been reported to cause adverse health effects in humans, especially neurological disorders including anxiety, depression, psychosis and peripheral neuropathy, as well as kidney dysfunction [1,2]. Monitoring the level of mercury ions (Hg^2+^) in cosmetic products is therefore of fundamental importance.

Due to mercury toxicity, in 1992, the United States Food and Drug Administration (US FDA) set a permissible value of mercury and its derivatives of less than 1 ppm. Many studies have been carried out to identify and quantify mercury in cosmetic preparations and have detected extremely high levels of mercury [3]. In Europe and China, the cap for cosmetic products for eyes and lips is 0.07 ppm. Likewise, Japan strictly prohibits the use of mercury in cosmetics. Terms to be searched for in the labeling of packaging include mercury, Hg, mercury iodide, mercury chloride, ammonized mercury and amide mercury chloride, among others. Instructions to avoid contact with silver, gold, rubber, aluminum and jewelry may also be an indication of the presence of mercury [4]. Nonetheless, mercury is not always listed as an ingredient in mercury-containing products.

The conventional analytical methods for the detection of Hg^2+^ usually rely on the use of advanced instruments based on spectrometric and chromatographic techniques, such as atomic absorption spectrometry (AAS) [1,5], inductively coupled plasma mass spectrometry (ICPMS) [6], graphite furnace atomic absorption spectrometry (GFAAS) [2], inductively coupled plasma atomic emission spectrometry (ICP-AES) [7] and liquid chromatography inductively coupled plasma mass spectrometry (LC-ICP-MS) [8]. While such approaches have been proven to be highly accurate and sensitive, the complex, expensive and time-consuming procedures associated with these methods call for a simpler alternative for daily analysis and on-site detection in a large number of samples in the field.

The electrochemical sensor has been an ideal tool for the detection of heavy metal ions, including Hg^2+^, due to its simplicity, quick response, low cost and portability. In electrochemical sensing, the type of working electrode is among the key factors that influence the analytical performance. Metal nanoparticles and carbon nanotubes have usually been used as working electrodes thanks to their large surface area and outstanding structural, electronic and optical properties [7]. The major limitation of an electrode made from precious metals is the high cost and complicated manufacturing. The carbon-based electrode, on the other hand, is cheap and easy to prepare but lacks stability and has poor reproducibility. Given its high optical clarity, good electrical conductivity and stable surface properties, indium tin oxide (ITO) film is of great interest as a cheap transparent electrode in the development of electrochemical sensors [9]. The bare ITO sensitivity for such an application, however, is not high. In this regard, modification of the ITO film is necessary. 

Composites made from a conductive polymer, such as polyaniline (PANI), and nanoparticles, such as multiwalled carbon nanotubes (MWCNTs) and gold nanoparticles (AuNPs), have been widely used in electrochemical sensors. The synergistic action between the high conductivity and stability of PANI [10], the high aspect ratio and exceptional mechanical strength of MWCNTs [11] and favorable electrocatalytic behavior of AuNPs could greatly enhance the sensor sensitivity and electroanalytical signals [12,13]. In this perspective, herein, for the first time, a PANI/MWCNT/AuNP-modified ITO electrode was developed by the direct electrodeposition of PANI, MWCNTs and AuNPs on a film-coated ITO electrode. The optimization of the electrode under various analyte conditions, interaction times and scan rates were performed, aiming to enhance the selectivity, sensitivity and reliability of the electrode for mercury detection. 

## 2. Materials and Methods

### 2.1. Chemicals and Reagents

All the reagents were of analytical grade and were used without further purifications. Tris(hydroxymethyl)aminomethane–HCl (Tris-HCl), MWCNTs, methylene blue (MB), gold nanoparticles (AuNPs), aniline, ITO and mercury standard solution were purchased from Sigma-Aldrich. All solutions and subsequent dilutions were prepared using deionized water from a filter instrument, namely Milli-Q (Millipark^®^ 40, Mount Holly, NJ, USA).

### 2.2. Instrumentations

**Figure 1:**
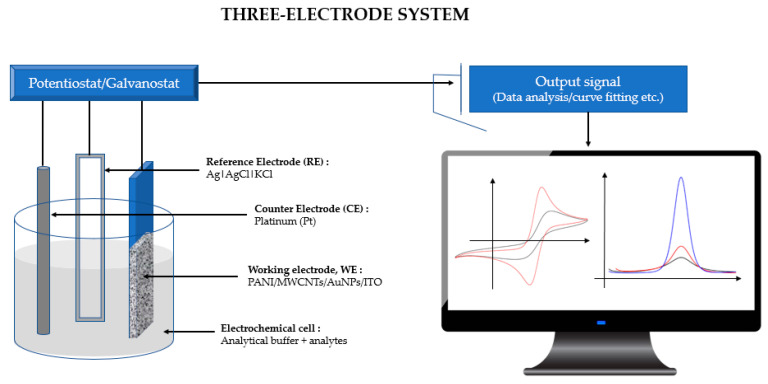
A Computer-controlled potentiostat/galvanostat with a standard three-electrode system connected to an electrochemical cell where Tris-HCl and mercury are used as analytes.

Electrochemical cyclic voltammetry (CV) and differential pulse voltammetry (DPV) measurements were recorded using an µ Autolab PGSTAT 30 computer-controlled potentiostat/ galvanostat (EcoChemie, Utrecht, The Netherlands) with a standard three-electrode system (Figure 1). The CV and DPV responses were subjected to statistical analysis using Tukey’s post hoc test. The modified ITO film-coated glass (Sigma-Aldrich, 60 Ω resistance) electrode (working area 1 cm × 1 cm) served as a working electrode, while platinum (Pt) was used as a counter electrode, with an Ag|AgCl|KCl 3 M reference electrode completing the cell assembly. All experiments were conducted in room temperature conditions of 23.0 ± 2.0 °C.

### 2.3. Electrode Preparation and Modification

The development and modification of the ITO electrode were performed using an electrochemical deposition technique following the method described by Tavakkoli, 2019 [14]. One milligram of MWCNTs was suspended in a 1 mL concentrated mixture of H_2_SO_4_ and HNO_3_ in a 3:1 ratio (v/v) and subjected to ultrasonication for 2 h to obtain a homogeneously distributed black solution. To remove acid, the functionalized MWCNTs were washed thoroughly with distilled water. To prepare PANI, 0.1 M ammonium persulfate was dropwise added to HCl, which contained a 0.1 M aniline solution, and it was kept for 12 h at room temperature. The deep green color of polyaniline was formed by a chemical oxidation process that was purified by centrifugation at 5000 rpm for 10 min. The mixture was stirred for 30 min, followed by sonication for 10 min for the formation of the nanocomposite. Briefly, the ITO flexible sheet was manually peeled off, followed by electrodeposition of 10 mg/mL PANI and 1 mg/mL MWCNTs and 0.02 g of oxidized AuNPs onto the ITO film. The ITO was dipped for 24 h into the mixtures. From the characterization, the combination of 10 mg/mL PANI and 1 mg/mL MWCNTs and 0.02 g of oxidized AuNPs formed a suitable nanocomposite as anticipated, and it was used for further sensing studies (50 mM Tris-HCl, pH 7.0). The resulting PANI/MWCNT/AuNP-modified ITO electrode was thoroughly washed with distilled water to remove unbound materials and kept at 4 °C in a dry Petri dish. The electrode surface was soaked in 1 mM of methylene blue (MB) for 2 min after effective immobilization, followed by washing with 50 mM Tris-HCl buffer (pH 7.0) to eliminate non-specific physical adsorption. 

### 2.4. Electrode Characterizations

The morphology of the modified ITO electrode was viewed by using SEM (Hitachi S-3400 N, Hitachi, Japan.). The electrode was fixed on the stub using carbon tape and sputter-coated with gold before the SEM observation. The analysis was performed using an accelerating voltage of 15 kV. Elemental analysis was performed by using energy dispersive X-ray spectroscopy attached to the SEM instrument. The Fourier infrared (FTIR) spectra were collected using an Agilent Cary 630 FTIR Spectrometer (Agilent Technologies Inc., Santa Clara, CA, USA) in the infrared region between 4000 and 600 cm^−1^ with a spectral resolution of 4 cm^−1^ and 32 scans at room temperature.

### 2.5. Optimization of Analyte and Operating Conditions

#### 2.5.1. Types of Redox Indicator

Methylene blue (MB, 1 mM) was prepared in a mixture of 50 mM Tris-HCl (pH 7.0) as a stock solution and diluted to the desired concentration using the same buffer. The electrochemical behavior of the modified ITO was characterized in the presence of MB. The electrode was first dipped in 2 mL MB for 2 min and rinsed with Tris-HCl buffer for 30 s to remove any non-specifically bound redox indicator. The electrode was then dried for 2 min at room temperature and transferred into an analytical buffer solution (50 mM Tris–HCl, pH 7.0) for cyclic voltammetry measurements. The signal was measured by scanning the potential from +0.0 to +2.0 V vs. Ag|AgCl at a scan rate of 0.10 V·s^−1^. The procedures were repeated by replacing MB with Prussian blue and Prussian red for the selection of the best redox indicator. The potential current of bare ITO with different types of redox indicator was compared. All experiments were conducted at room temperature unless otherwise stated.

#### 2.5.2. Types of Analytical Buffer

Four types of the buffer, including Tris-HCl, phosphate, acetate and citrate buffers, were investigated as the electrolytic solution. The buffers were prepared in 10 mL with a concentration of 0.1 M in the presence of mercury. 

#### 2.5.3. The pH of the Analytical Buffer

The effect of pH was determined in the range of pH 6.0–8.0 in a solution containing 6.0 ppm standard mercury in the buffer.

#### 2.5.4. Incubation Times

The electrochemical behavior of the modified ITO was characterized by CV using a redox indicator in a solution containing 6.0 ppm standard mercury and buffer with different incubation times (5, 10, 20, 30, 40, 50, 60, 70, 80 s). 

#### 2.5.5. Scan Rates

The study was done by adjusting the scan rate in the range of 0.01–0.30 V·s^−1^ in the electrochemical system containing analytical buffers with 6.0 ppm standard mercury. 

### 2.6. Detection of Raw Samples

Samples of the cosmetic products were obtained from a local store in Kota Kinabalu, Sabah, Malaysia for analysis purposes. First, 1.0 g of sample was added into a centrifuge tube containing 2.0 mL deionized water and ultrasonicated for 5 min to facilitate the complete dissolution of mercury and subsequently filtered using filter paper (Whatman no. 541). This solution served as the stock solution for the preparation of the samples for analysis. An appropriate volume of aliquot was taken from the clear part of the solution and transferred into the electrochemical cells. The volume of the working solution was filled to 10 mL by Tris-HCl buffer (50 mM, pH 7.0). Suitable aliquots of this solution were analyzed by the proposed method for mercury determination [15,16]. Finally, the pretreated sample was analyzed using cyclic voltammetry (CV) and differential pulse voltammetry (DPV) under the optimal conditions at +0.4 to +2.0 V, as defined by the potentiostat/galvanostat.

## 3. Results and Discussion

### 3.1. Characterization of PANI/MWCNTs/AuNPs/ITO

The morphological characterization of the modified ITO electrode was examined by a scanning electron microscope (SEM). Meanwhile, energy-dispersive X-ray (EDX) and Fourier transform infrared spectroscopy (FTIR) were used to analyze the functional groups and elements present in the electrode.

**Figure 2:**
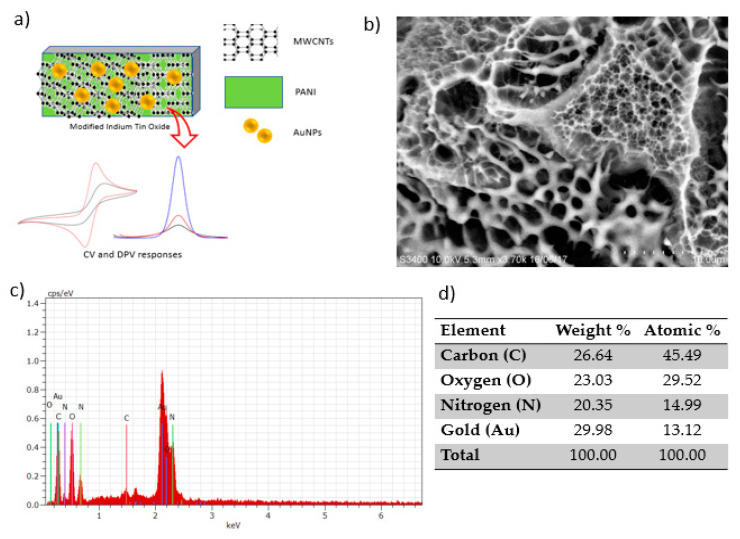
Polyaniline/multiwalled carbon nanotube/gold nanoparticle (PANI/MWCNT/AuNP)-modified indium tin oxide (ITO) electrode: (**a**) Schematic representation, (**b**) SEM image, (**c**) energy-dispersive X-ray (EDX) spectra and (**d**) the corresponding elemental composition.

Figure 2a shows the illustration of ITO electrode modification by the electrodeposition of PANI, MWCNTs and AuNPs. The morphological structure of the homogenous surface of the modified electrode viewed under SEM is shown in Figure 2b. AuNPs can be seen with a smooth and featureless morphology as bright components well distributed in the MWCNT and PANI network [17]. The figure shows the granular morphology of PANI. The carbon nanotubes are properly embedded in the PANI matrix due to interfacial bonding between MWCNTs and the PANI matrix. The greater amount of PANI covers MWCNTs and forms the PANI/MWCNT composite [11]. Such a structure could afford a high effective surface area for the adsorption and diffusion of mercury, thus improving the sensitivity of electrochemical sensors [18]. The corresponding elemental analysis of the modified electrode clearly shows the presence of nitrogen (N), oxygen (O) and carbon (C), and gold (Au) elements in EDX spectra confirmed the successful attachment of the PANI and MWCNTs onto the ITO electrode (Figure 2d). 

The interactions between PANI and carboxyl functional MWCNTs take place due to electrostatic interactions between –COO^−^ groups of MWCNTs and the –NH^+^ of the aniline monomer, the hydrogen bonding between –OH groups on MWCNTs and the –NH group of the aniline monomer, and also a small amount of –π staking between the π-bonds of the MWCNTs and the quinoid ring of the aniline monomer [11]. Such a strong interaction ensures that the aniline monomer is absorbed on the surface of the MWCNTs which serve as the core and the self-assembly template (deposition) during the formation of tubular nanocomposites. When the aniline monomers are mixed with AuNPs, the oxidation of aniline monomers occur simultaneously, leading to in situ polymerization cause by strong interactions between MWCNTs and aniline monomers. Although there are carboxylic groups on the defect sites of MWCNTs to increase the solubility of MWCNTs in HCl solution, some MWCNT bundles remain randomly configured. Consequently, some gaps may exist between individual MWCNTs to allow the aniline monomers to wriggle into such gaps, followed by in situ polymerization caused by the strong interaction between MWCNTs and aniline monomers. 

The growing PANI polymer chain would wedge away from the MWCNT bundles and then break down the bundles into individual MWCNTs. In this case, MWCNTs are uniformly and individually dispersed in PANI matrices. The site-selective interaction between the quinoid ring of the polymer and MWCNTs caused PANI polymer chains to be adsorbed at the surface of the MWCNTs and form the shell of the tubular nanocomposites [19]. PANI is one of the best-known conductive polymers with favorable structural and chemical versatility. Its NH_2_^−^ enriched chemical backbone offers the flexibility of binding with various molecules [10]. PANI templating by MWCNTs provided a head-to-tail structure under the optimized condition. When AuNPs are added, the intermolecular hydrogen bonds are weak and new hydrogen bonds are formed between PANI and MWCNTs, which facilitate the rotation and movement of the molecular chain [11,20]. 

**Figure 3:**
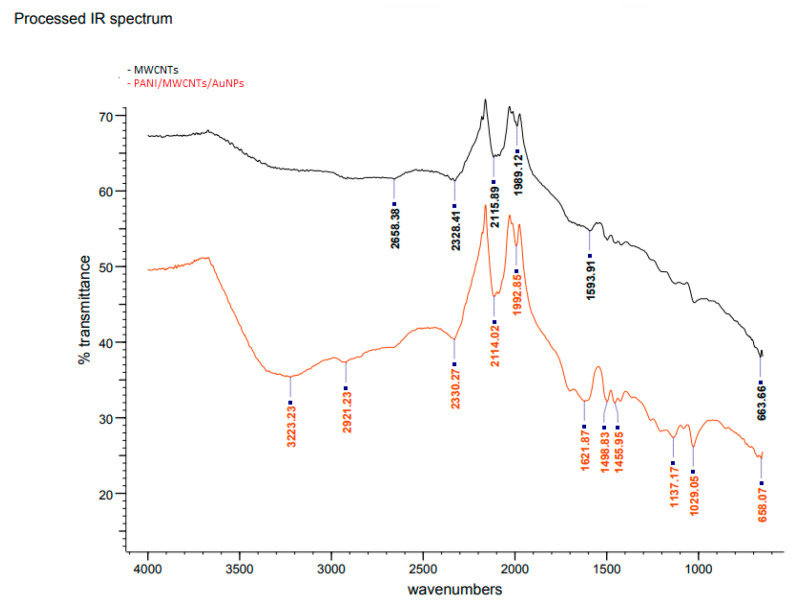
FTIR spectra of bare and modified ITO electrodes.

Figure 3 shows the FTIR spectra of MWCNTs (Curve I) and PANI/MWCNT/AuNP electrodes (Curve II). The bands at 2658, 2328, 2115, 1989 and 1593 cm^−1^ are characteristic of MWCNTs. These bands are slightly shifted in the modified ITO electrode, with the presence of several new absorption bands particularly at the fingerprint region, which is a clear indication of the modification of the electrode due to the presence of PANI, MWCNTs and AuNPs. Accordingly, the broad peak at 3223 cm^−1^ could be assigned to the O–H stretching vibrations of carboxylic groups (O=C–OH). These two peaks are an indication of acid functionalization. Meanwhile, the peak at 2921 cm^−1^ is for –CH_2_ antisymmetric stretching, which is produced at the defect sites of acid-oxidized MWCNT surfaces [21]. Hence, there is no stretching at 1700 cm^−1^ which is supposedly assigned as a carboxylic acid. The absorption band at 1621 cm^−1^ is assigned to N-H stretching of the amides since it is medium intensity. For PANI-MWCNTs, the main peaks at 1455, 1137 and 658 cm^−1^ belong to the C=C stretching of the benzenoid ring and quinoid ring, C–N stretching of the secondary aromatic amine and C–S–C symmetric, respectively [22]. The absorption band at 2330 cm^−1^ and 2114 cm^−1^ is attributed to the stretching vibration of C=N, meanwhile, bands at 1498.83 cm^−1^ and 1455.96 cm^−1^ are assigned to –C–N stretching of 2° amines of quinoid and benzenoid structures of PANI due to polymerization of aniline to form a PANI chain linked through –NH– bonds of Au with two free –NH_2_ group at its ends [11]. The peak of the C–O bond of the free –COOH group is present at 1029 cm^−1^. It reveals that the –COOH group at one end of the MWCNT gets attached to the –NH_2_ group of PANI (at the end of its chain) through a –CO–NH– bond, as reported by Patel et al. (2020) [23]. 

**Figure 4:**
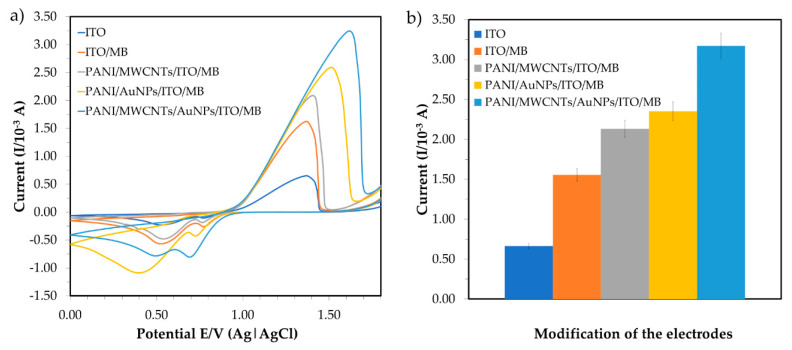
Comparison of electrochemical behavior for bare ITO, PANI/MWCNTs/ITO, PANI/AuNPs/ITO and PANI/MWCNTs/AuNPs/ITO in the presence of methylene blue (MB) as a redox indicator; (**a**) cyclic voltammogram and (**b**) the corresponding peak current.

Cyclic voltammetry (CV) is used for determining the specificity of the modifications, as shown in Figure 4a. In the presence of 6 ppm mercury, the redox signals of the accumulated MB on the ITO surfaces were explored with potentials ranging from +0.0 to +1.8 V and a scan rate of 0.10 V·s^−1^. Karpuraranjith and Thambidurai (2016) [24] reported that PANI and MB act as efficient binding agents in electrochemical sensors. MB is an important source of increment ion carriers in nanomaterials for transporting charges. Due to the combination of nanoparticles and nanomaterials, the conductivity efficiency of the sensor comprising PANI/MWCNTs/AuNPs is significantly enhanced in the potential current. The MWCNT N-alkyl chain length is due to van der Waals interactions between nanoparticles (AuNPs) and nanocomposites (MWCNTs). The comparison of peak currents for bare ITO, PANI/MWCNTs/ITO, PANI/AuNPs/ITO and PANI/MWCNTs/AuNPs/ITO in the presence of MB as a redox indicator is shown in Figure 4. The peak currents increased due to the dual charges [25] of MB. By substituting MWCNTs with AuNPs, a higher peak current was observed for the PANI/AuNP/ITO electrode compared to the PANI/MWCNT/ITO electrode signal. The modified ITO potential was shifted to the right and the potential separation was larger than that of the bare ITO. Additionally, the current signals of PANI/MWCNTs/ITO (1.596 × 10^−3^ A) were larger than the bare ITO signals (1.015 × 10^−3^ A) (Figure 4b). 

AuNPs are conductive components that are used as a base to change the working electrode that will amalgamate the mercury. AuNP assembly on PANI is uniform and showed excellent features, such as high catalytic activity because of high surface modification area (nano size) and effective electron transport for electrochemical sensors [18]. The peak current was much higher when the ITO was modified with PANI/MWCNTs/AuNPs and peak voltage was shifted from +1.43 to +1.56 V. This indicated that the introduction of the MWCNTs/AuNPs played a vital role in increasing the electroactive surface area and providing the conducting bridges for the electron transfer of MB. Nanoparticles are then attached to MWCNTs and the PANI network matrix and increase in surface area to improve the sensitivity for mercury detection. Since PANI can dissolve many transition metal complexes, it is used to increase electrochemical sensor reaction rates and selectivity [22]. Regularly, the increasing oxidation peak currents indicated that the combination of MWCNTs/AuNPs attached to the PANI nanocomposite produced highly conductive materials that were persistently deposited on the ITO. Hence, PANI/MWCNTs/AuNPs were effectively immobilized onto the ITO surface in the presence of MB and provided the necessary pathways of conduction for the determination of mercury.

### 3.2. Optimum Conditions for Mercury Detection

#### 3.2.1. Effect of the Redox Indicator

**Figure 5:**
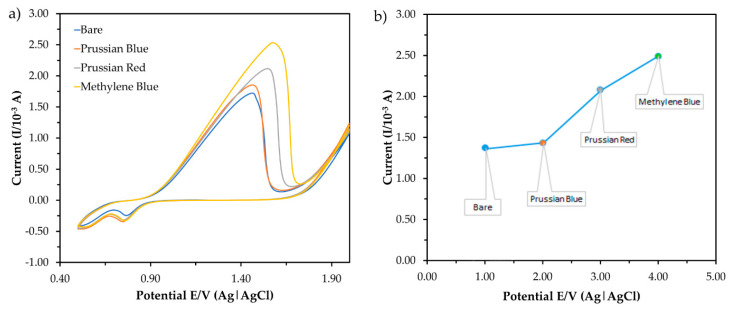
The current response obtained from the measurement of three (3) types of redox indicator in (**a**) cyclic voltammetry (CV) and (**b**) the corresponding peak current in the presence of mercury in the electrolytic solution (50 mM Tris-HCl buffer, pH 7.0).

Figure 5 shows the current responses obtained from the measurement of three types of redox indicators in the CV. The concentration of the buffer and pH was set at 1 mM and pH 7.0, respectively, for this measurement. Then, 1 mM methylene blue (MB) was chosen as the redox indicator which gave the highest peak current. MB is a heterocyclic chemical compound that can enhance the electron transfer in electrochemical sensors as a redox indicator [25]. MB presents a well-defined two-electron redox process with direct signaling cathodic and anodic peaks. The peak currents were measured with MB and without MB in the potential cycling, ranging from +0.0 to +1.8 V versus E/V (Ag) at a scan rate of 0.10 Vs^−1^ with bare ITO. At a potential value of +1.3 V, the oxidation current signal of the bare electrode in the presence of MB (1.0 × 10^−3^ A) is higher than for the electrode in the absence of MB (4.4 × 10^−4^ A). Due to the large amounts of electron transfer in the electrochemical cells, the redox peak current was significantly increased after MB was added. The current signals were used to monitor the process, which corresponded to the MB reduction. This process increased the electron transfer to the ITO and the current-produced intensity. According to a previous study reported by Fu et al. (2017) [26], the MB was decreased at the ITO by two reactions of electron transfer. The mercury was attended in the system based on the results which caused oxidation of –NH_2_. The MB is recycled as the mediator of the ITO which leads to the increase in current production. Thus, 1 mM MB was accumulated in the ITO for further parameter studies.

#### 3.2.2. Effect of Different Types and pH of Buffer Solution

**Figure 6:**
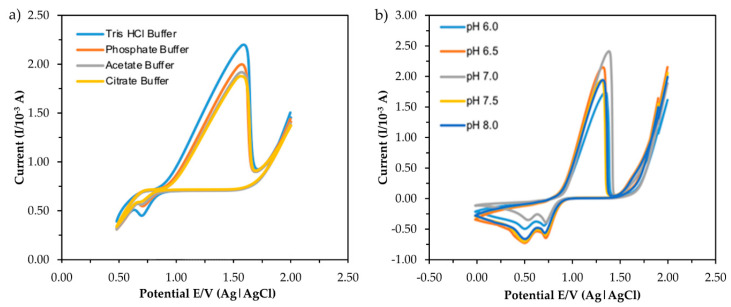
CV responses of different type of buffer (**a**) and pH (**b**) in the presence of 6 ppm mercury.

The current response obtained from the CV measurement of five types of buffer and different pH is shown in Figure 6. For investigating the effect of buffer type, the buffer concentration and pH were fixed at 0.1 M and pH 7.0, respectively, while 1 mM MB was used as a redox indicator. The buffer provides a complete circuit by acting as an electrolytic solution. The volume of the electrolytic solution was fixed at 10 mL with the presence of 6 ppm standard mercury. The sharp response peaks at the potential range in both indicate that the process was diffusion controlled [27,28]. The highest anodic peak current of mercury interaction was reached using Tris-HCl buffer compared to other types of the buffer, suggesting the favorable nature of the buffer towards the electroanalytical signal. Thus, the pH of the Tris-HCl buffer was then varied from 6.0 to 8.0 to investigate the effect of pH on the CV response.

As can be seen in Figure 6b, the highest anodic peak current was obtained at neutral pH (pH 7.0) and the peak was slightly shifted to a higher potential, which was a consequence of the oxidation cycle involving a higher transfer of protons (H^+^). The results indicate that mercury oxidation on ITO requires different numbers of H^+^ or OH^−^ transferred at different pH values, resulting in different electrochemical responses, as Roushani et al. (2017) reported [29]. The decrease in response at pH 7.0 could be due to the hydrolysis of some mercury ions in the solution, which later prevented ions from forming a temporary bond with the surface of the electrode [16] By considering the response and behavior of the electrochemical sensor, pH 7.0 was selected as the optimum pH for another experimental study.

#### 3.2.3. Effect of Incubation Times

**Figure 7:**
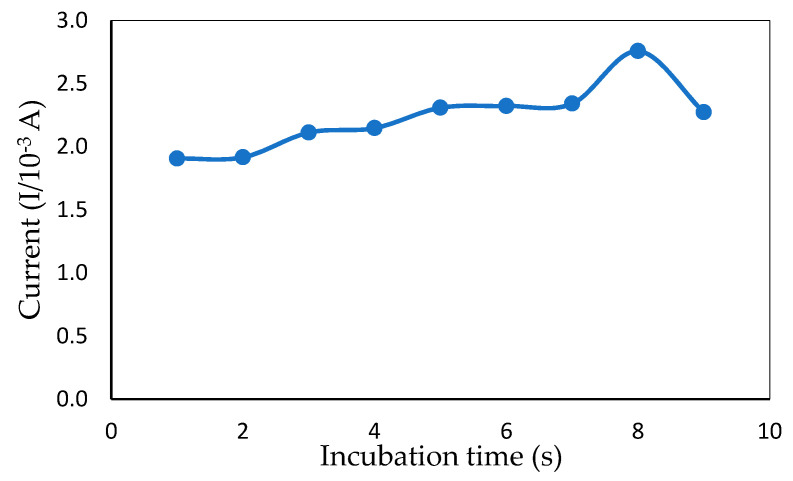
Peak current obtained from CV measurement showing the effect of different incubation times in the presence of 1 mM MB and 6 ppm of mercury (50 mM Tris-HCl buffer, pH 7.0).

Changing the time of incubation between ITO and mercury is a simple, effective way of varying sensor electrochemical sensitivity [30]. The time of incubation for mercury in the presence of a redox indicator affected the efficiency of the ITO. The CV incubation time between ITO and mercury (6 ppm) from 5–80 s is shown in Figure 7. A rapid increase in the peak current response was observed from 5 to 70 s. After 70 s, the current response dropped, which could have been because the interface of the modified electrode was fully occupied by mercury and could not bind after a certain amount of time with other molecules. Thus, 70 s was selected as the optimum incubation time for rapid mercury detection.

#### 3.2.4. Effects of Scan Rate

**Figure 8:**
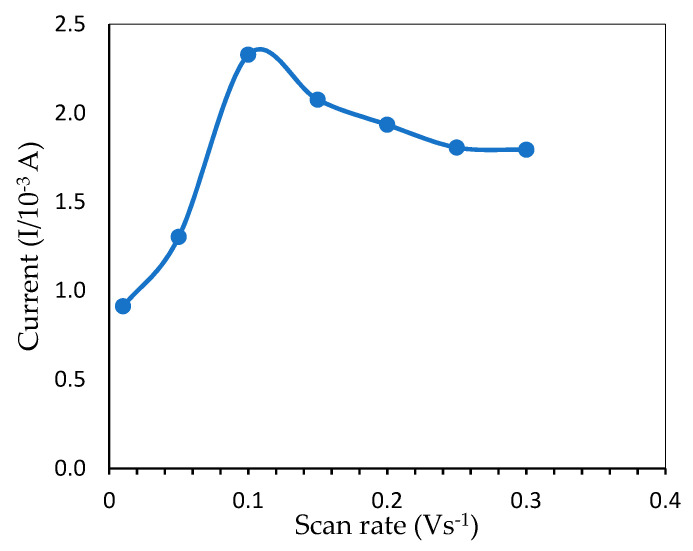
Peak current from CV measurement showing the effect of different scan rates for the determination of 6 ppm mercury (pH 7.0, 50 mM Tris-HCl buffer).

The relationship between peak current and scan rate is a piece of useful information related to the electrochemical mechanism. To avoid overoxidation, the influence of the potential scan rate on mercury electrochemical oxidation was investigated within 0.01–0.30 V·s^−1^, as shown in Figure 8. Initially, the peak current increased when the scan rate was adjusted from 0.01 to 0.10 V·s^−1^ and reached a maximum value due to the increased kinetics of electron transfer on the electrode surface [30]. However, as the scan rate increased even further until 0.30 V·s^−1^, the oxidation wave became unstable, presumably due to overoxidation by the ITO [31] and caused the peak current to drop gradually, indicating that mercury redox onto ITO was a surface-controlled operation. As a result, 0.10 V·s^−1^ was selected as the optimum scan rate which gave the highest oxidation peak.

### 3.3. Electrochemical Analysis of Modified ITO

#### 3.3.1. Reproducibility and Repeatability

**Figure 9:**
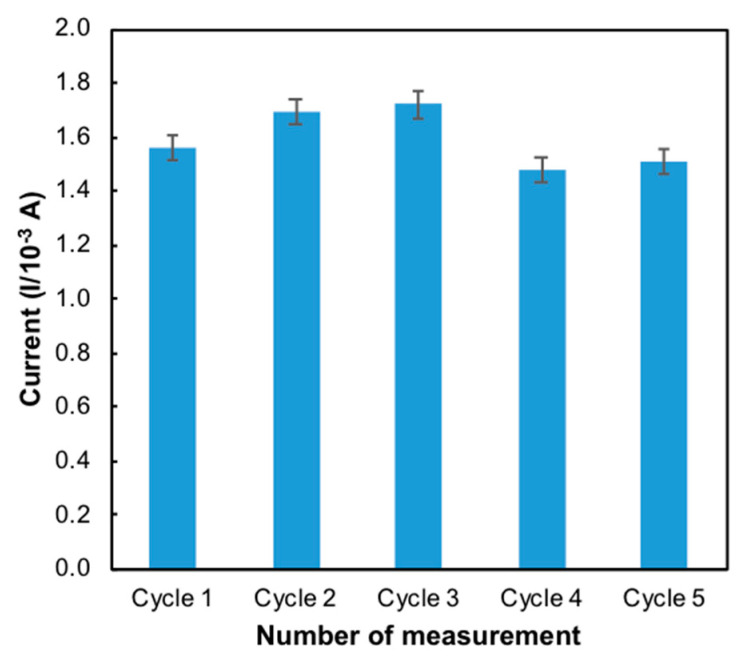
Bar chart of the reproducibility of the developed sensor (*n = 5*).

Reproducibility and repeatability are the two extremes of precision. Repeatability describes minimum variability and reproducibility the maximum variability in results. One of the important characters of a sensor is its reproducibility. Five independent modified electrodes were prepared on the same day and under the same conditions (50 mM Tris-HCl, pH 7.0, 1 mM MB) to investigate the reproducibility. It was evaluated with the same mercury concentration on five consecutive days with five modified electrodes independently prepared in the same experimental conditions. The reproducibility of the electrode was 1.69 × 10^−4^ ± 2.31 × 10^−6^ (Figure 9) with a relative standard deviation (RSD) of 2.82% (Table 1). Repeatability essentially depends on the stability of the nanomaterials that attached to the surface of the electrode [32]. The same process of determination of mercury on the same day was repeated using five modified electrodes under the same conditions. [33] The RSD of repeatability for the developed sensor was found to be 1.24%. These results indicated that the modified ITO electrode possesses good reproducibility and acceptable repeatability.

#### 3.3.2. Interference Studies

**Figure 10:**
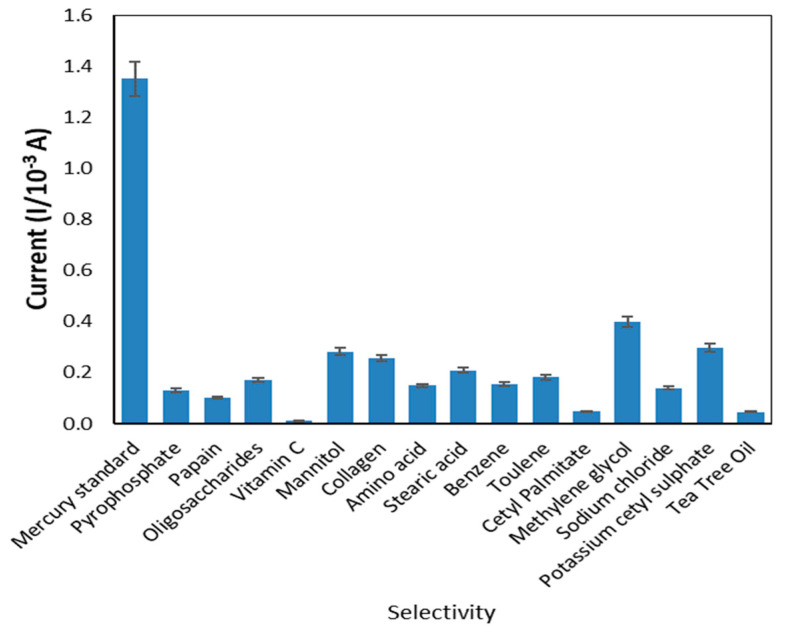
The selectivity of the developed sensor.

In the presence of certain metal ions and compounds commonly found in cosmetic products, such as pyrophosphate, papain, oligosaccharides, vitamin C, mannitol, collagen, amino acid, stearic acid, benzene, toluene, cetyl palmitate, methylene glycol, sodium chloride, potassium cetyl sulphate and tea tree oil [34,35], the current responses were analyzed to verify the selectivity of the sensor created. The metal ions and other elements from the literature have the potential to affect the condition. Figure 10 indicates the peak currents produced from reactions of the modified electrode in the presence of mercury and common disrupting compounds in cosmetics. The peak current of mercury was substantially higher than that of other compounds, indicating that the sensor exhibited greater selectivity for mercury compared to other compounds. 

#### 3.3.3. Limit of Detection (LOD)

**Figure 11:**
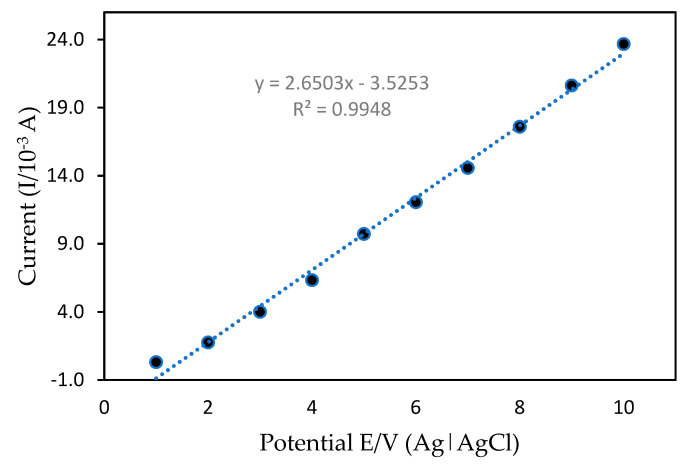
Calibration curve between the current peak of differential pulse voltammetry (DPV) and the potential.

The calibration graph and mercury detection limits of the developed mercury sensor were analyzed using the DPV method. DPV is a sensitive technique which allows for the high accuracy determination of the analyte in a relatively short period. Figure 11 shows the calibration curve of mercury in 50 mM Tris-HCl (pH 7.0) with 1 mM MB as a redox indicator with a possible range of +0.4 to +2.0 V using a 0.10 Vs^−1^ scan rate. With different mercury concentrations in the range of 0.1–10.0 ppm and a detection limit (based on Equations (1) and (2)), the anodic peak current was increased proportionally under optimization conditions. The linear regression equation with a correlation value of R^2^ = 0.9969 can be expressed as *y* = 1.174*x* − 1.3327. The mean value obtained from independent measurements corresponded to the calibration graph for each point. These findings are due to the mercury interactions with PANI/MWCNTs/AuNPs at the ITO surface. More importantly, modification of the ITO enabled the detection of trace amounts of mercury with concentrations as low as 0.03 ppm, suggesting high sensitivity of the method as compared to other methods that have been reported previously (Table 2). The low detection limit obtained here could be attributed to the strong adsorption ability and large specific PANI/MWCNT/AuNP/ITO surface area that enhanced mercury electron transfers with modified ITO and excellent nanocomposite interactions.

(1) $$LOD=3\frac{S}{m}$$

(2) $$Sensitivity=\;\frac{m}{A}$$

where *S:* Standard deviation of the blank measurements, *m:* Slope of the calibration curve, *A:* Area of the electrode.

#### 3.3.4. Analytical Application on Cosmetic Products

The developed sensor was applied to analyze mercury in cosmetic products bought from a local store in Kota Kinabalu, Sabah, Malaysia to confirm the sensitivity and performance of the proposed method. The samples were then measured using the DPV method under the optimum conditions. In Table 3, about 89% of the cosmetics contained mercury concentrations below detection (<0.01 ppm) and below the 1 ppm safety limit set by the FDA. The highest concentration of mercury was recorded in the Dye of Haircare product sample, 2.129 ± 0.057 ppm, 2.135 ± 0.085 ppm, and 1.060 ± 0.039 ppm in the Foundation of Decorative cosmetic. All the skincare, body care and perfumes analyzed had concentrations below 0.01 ppm. These products were obtained from the local market, and originated from Asian countries, such as the Philippines, Thailand, Cambodia and China. A good recovery rate (96.6–97.5%) and relative standard deviation (RSD) (<1%) were obtained, as indicated in Table 4. According to Mathias et al. (2014) [36], a recovery percentage of 80% is an indication of high accuracy. Equation (3) was used to calculate the percentage of recovery. The amount of mercury measured in the cosmetic product samples met the normal requirement. With a high sensitivity, a wide linear range and a low detection limit, the proposed method holds great promise for determining mercury in cosmetics.

(3) $$Percent\;of\;recovery=100\times \frac{C_{spiked\;sample}\;-C_{unspiked\;sample}}{C_{added}}$$

## 4. Conclusions

In this study, the modification of ITO using PANI, MWCNTs and AuNPs was used for the determination of mercury. The characteristic features of the PANI/MWCNT/AuNP composite were studied by using SEM, EDX and FTIR. The optimization of the modified electrode for mercury determination to formulate a nanocomposite was an appealing factor to elevate the sensing performance. This modified electrode for mercury sensors performed under optimum buffer conditions, redox indicator, pH, scan rate and incubation time and allowed for a rapid detection of the target ion in 70 s with a satisfactory current response. The optimization method permitted the detection of mercury ions at various concentrations, while measuring the linear range from 0.01 to 10.0 ppm. The developed sensing platform was highly sensitive and selective toward aqueous ammonia among the pool of possible interferents. The linear responses for different concentrations of mercury ranged of 0.01–10.0 ppm with a high correlation coefficient (R^2^ = 0.9969) and a limit of detection (LOD) of 0.03 ppm of the nanocomposite, which gave a platform for the sensitive detection of mercury in real sample analysis. This PANI/MWCNT/AuNP composite is easily reproducible and has high repeatability. The present approach extended a new path for the selective and sensitive detection of mercury, which is essential in biomedical, environmental monitoring and chemical industry fields in real time. 

## Figures and Tables

**Table 1:** Reproducibility and repeatability analysis of the modified electrode.

Characteristic	Mean ± STD	RSD (%)
**Reproducibility**	1.69 × 10^−4^ ± 2.31 × 10^−6^	2.82
**Repeatability**	1.48 × 10^−4^ ± 3.54 × 10^−6^	1.24

**Table 2:** Comparison with other sensor methods for determination of mercury.

Type of Sensor	Sensing Principle	Detection Limit (mol L^−1^)	Reference
Electrochemical sensor	Interaction with nanomaterials and nanoparticles	1.5 × 10^−7^ mol L^−1^ (0.03 ppm)	This study
Electrochemical sensor	Interaction with nanoparticles and folic acid	8.43 × 10^−6^ mol L^−1^	[32]
Fluorescent optical fiber chemosensor	Photoinduced electron transfer	~10^−6^ mol L^−1^	[33]
Colorimetric sensor	Formation of the colored complex by dithizone ligand	1.59 × 10^−4^ mol L^−1^	[34]

**Table 3:** Determination of mercury concentration in cosmetic samples.

Cosmetic Products	Forms and Properties	Raw Samples	Mean ± STD
Sample 1 Skincare	Lotion	Sample 1 a	<0.01
	Serum	Sample 1 b	<0.01
	Moisturizer	Sample 1 c	<0.01
Sample 2 Suncare	Cream	Sample 2 a	0.075 ± 0.002
	Lotion	Sample 2 b	<0.01
	Gel	Sample 2 c	<0.01
Sample 3 Haircare	Hair straightener	Sample 3 a	0.059 ± 0.012
	Shampoo	Sample 3 b	<0.01
	Dye	Sample 3 c	2.129 ± 0.057
Sample 4 Body care	Soap	Sample 4 a	<0.01
	Oil	Sample 4 b	<0.01
	Shower gel	Sample 4 c	<0.01
Sample 5 Decorative cosmetic	Face powder	Sample 5 a	0.106 ± 0.028
	Foundation	Sample 5 b	2.135 ± 0.085
	Lipstick	Sample 5 c	1.060 ± 0.039
Sample 6 Perfumes	Scented oil	Sample 6 a	<0.01
	Deodorant	Sample 6 b	<0.01
	Salve	Sample 6 c	<0.01

**Table 4:** Recovery study of the developed sensor.

Developed Sensor	**Added Concentration (ppm)**	**Found Concentration (ppm)** Mean ± STD *	**Recovery (%)**	**RSD (%)**
	0.03 ppm	0.03 ± 0.38	96.6%	0.43%
	6 ppm	5.7 ± 0.45	97.5%	0.52%
	10 ppm	9.49 ± 0.43	97.3%	0.64%

* Mean value of five replicates ± standard deviation (*n* = 5).
